# Hot and scared: how do heatwaves and predation risk impact resource acquisition and allocation?

**DOI:** 10.1098/rsbl.2024.0009

**Published:** 2024-04-24

**Authors:** Zachary R. Stahlschmidt, Harnoor Joura, Jenna R. Makarem, Jodie L. Sun

**Affiliations:** ^1^ Department of Biological Sciences, University of the Pacific, Stockton, CA, USA

**Keywords:** cricket, decision making, environmental change, life history, trade-off

## Abstract

Heatwaves are increasingly prevalent and can constrain investment into important life-history traits. In addition to heatwaves, animals regularly encounter threats from other organisms in their environments, such as predators. The combination of these two environmental factors introduces a decision-making conflict—heat exposure requires more food intake to fuel investment into fitness-related traits, but foraging in the presence of predators increases the threat of mortality. Thus, we used female variable field crickets (*Gryllus lineaticeps*) to investigate the effects of heatwaves in conjunction with predation risk (exposed food and water sources, and exposure to scent from black widow spiders, *Latrodectus hesperus*) on resource acquisition (food intake) and allocation (investment into ovarian and somatic tissues). A simulated heatwave increased food intake and the allocation of resources to reproductive investment. Crickets exposed to high predation risk reduced food intake, but they were able to maintain reproductive investment at an expense to investment into somatic tissue. Thus, heatwaves and predation risk deprioritized investment into self-maintenance, which may impair key physiological processes. This study is an important step towards understanding the ecology of fear in a warming world.

## 1. Introduction

Heatwave events are expected to become more frequent and intense [1], and warmer temperatures obligate increased energy expenditure for most animals [2]. Thus, animals’ reliance on food is linked to heatwaves—increased food consumption can offset the metabolic costs of elevated temperatures and promote heat tolerance [3–5]. However, foraging entails predation risks for many animals [6,7], and elevated temperatures can increase predation through improved capture efficiency and shifts in the functional response [8–10]. Therefore, foraging decision making plays a fundamental role in the ecology of fear—whereby the sub-lethal, fitness-related costs of avoiding predation may exceed the population-level costs of predation to prey [11,12]—in a warming world.

Animals naturally vary in their acquisition of food, which can strongly influence investment into important life-history traits [13–16]. The classic life-history trade-off between soma (storage and self-maintenance) and reproduction can be eliminated when food is abundant [17–19]. Furthermore, large inter-individual variation in resource acquisition can even result in positive covariation between traits [13]. Thus, it is important to account for food consumption when determining plasticity in life-history strategies, which may be one of the animals’ key defences against ongoing environmental change [20–22]. Warming influences resource allocation patterns across animal taxa [23–25]—yet, the combined and potentially opposing effects of heatwave exposure and predation risk on life-history strategy are poorly understood.

Here, we disentangled the roles of resource (food) acquisition, heatwaves and predation risk on life-history trait investment and resource allocation strategies. In the variable field cricket (*Gryllus lineaticeps*), we factorially manipulated predation risk (sheltered food and an absence of predator cues versus exposed food and presence of predator cues) and temperature (control regime versus simulated heatwave) to address three aims. In *G. lineaticeps*, heatwave exposure increases feeding and resource allocation to reproductive tissue [26], *sensu* a ‘terminal investment’ strategy where stress leads to the prioritization of current reproduction to maximize lifetime reproduction (reviewed in [27]). The first aim addressed whether predation risk similarly facilitates terminal investment or, by contrast, a reproductive restraint strategy where stress prioritizes somatic investment over current reproduction, presumably to facilitate future reproductive opportunities (reviewed in [28]). Whether an organism adopts a terminal investment or reproductive restraint strategy in response to an immune challenge can depend on intrinsic and extrinsic factors, including age and threat level, respectively (reviewed in [27]). Therefore, the second aim addressed whether the expression of terminal investment or reproductive restraint was similarly context-dependent—specifically, if the effects of heatwave and predation risk interact with one another to influence life-history strategy. For example, predation risk may favour reproductive restraint in control temperature conditions, but terminal investment in heatwave conditions. The third aim addressed the role that resource acquisition plays in mediating the plasticity of life-history investment strategies in response to heat and predation risk. Here, we tested whether heatwave and predation risk exerted (i) direct effects on life-history strategy, or (ii) solely indirect effects that were mediated via changes in feeding. Through these aims, we integrate multi-trophic interactions and climate change to better understand the plasticity of life-history strategies.

## 2. Methods

### (a). Study system

The variable field cricket (*G. lineaticeps*) is found from southwestern Oregon, through California, and into Baja California, Mexico [29]. We only used short-winged (SW) crickets in the study, and we acquired them from a long-term colony that was subsidized every 1–2 years by progeny from females collected from a natural population (Sedgwick Reserve, Santa Ynez, CA) that predominately expresses the short-winged phenotype (ZR Stahlschmidt 2018, personal observation; LA Treidel 2022, personal communication). We reared *G. lineaticeps* in standard conditions (14 L : 10 D cycle with ad libitum access to water, commercial dry cat food and cardboard egg cartons for shelter) at 28 ± 1°C. Within 1 day of their adult moult, we weighed and individually housed females in 15 l transparent plastic containers containing pre-weighed food (dry cat food pellets). We then assigned crickets to one of four environmental treatment combinations (electronic supplementary material, figure S1).

### (b) Experimental design

We used a 2 × 2 factorial design to study the independent and interactive effects of environmental treatment (predation risk and temperature) on resource acquisition and allocation. We examined these dynamics during the first 6 days of adulthood because this period is characterized by intense resource acquisition and allocation in *Gryllus* crickets—SW (short-winged) females can increase ovary mass by 100-fold or more [30,31]. *Gryllus* adults typically live only 2 weeks or less in the field [32], meaning our 6 days study period spanned approximately half of the crickets’ natural lifespan. We randomly assigned half of the females (*n* = 68) to a treatment simulating high predation risk by forcing them to eat and drink in open, exposed locations and by exposing them to scent from a sympatric predator, the western black widow spider (*Latrodectus hesperus;* electronic supplementary material, figure S1). We assigned the remaining crickets (*n* = 63) to a low predation risk treatment characterized by sheltered food and drinking water, and by a lack of predator scent (electronic supplementary material, figure S1). We also randomly assigned half of the crickets (*n* = 69) to a control temperature treatment (17–31°C daily cycle) and the remaining crickets (*n* = 61) to a simulated heatwave treatment (24–38°C daily cycle) as described previously ([26]; electronic supplementary material).

After 5 days, we reweighed each cricket and its food. We then euthanized and stored each cricket at −20°C before estimating investment into and allocation to reproductive and non-reproductive (somatic) tissues as previously described [26,33]. Briefly, after storage, we removed and weighed each cricket’s ovaries to determine total investment into reproduction. We first estimated initial (day 1) ovary mass (i.e. 0.98% of day 1 total body mass [34]; electronic supplementary material). Then, we estimated the amount of ovary mass gained during the experiment (total reproductive investment) and, by subtraction, the amount of somatic tissue mass gained (total somatic investment) based on the total body mass gained (electronic supplementary material). Next, we used the amount of food consumed during the experiment to estimate the efficiency by which ingested food was converted into ovary mass (allocation to reproduction) and somatic tissue mass (allocation to soma) (electronic supplementary material).

### (c). Statistical analyses

We tested data for normality, and we analysed data using SPSS (v.28 IBM Corp., Armonk, NY) and jamovi (v.2.3; PATHj, based on the R package lavaan [35]). We determined two-tailed significance at *α* = 0.05. To examine the independent and interactive effects of environmental treatments (predation risk and temperature), we performed linear mixed model analyses on (i) the amount of food consumed, as well as the estimated mass of (ii) reproductive and (iii) somatic tissues gained, and conversion efficiencies for (iv) reproductive and (v) somatic tissues. For these models, we included environmental treatments as the main effects and initial (day 1) body mass as a covariate to account for differences in body size. We report significant results below, and full results can be found in the electronic supplementary material, tables S1–S5. To examine complex dynamics (e.g. the trade-off between investment into reproductive versus somatic tissues) and visualize the relative weights of each effect (*sensu* [36]), we used path analyses on (i) total investment (reproductive and somatic tissue mass gained), and (ii) resource allocation (conversion efficiencies for reproductive and somatic tissues) as previously described ([33] electronic supplementary material). We report full statistics for both path analyses’ initial and final models in the electronic supplementary material, tables S6 and S7.

## 3. Results

**Figure 1.**
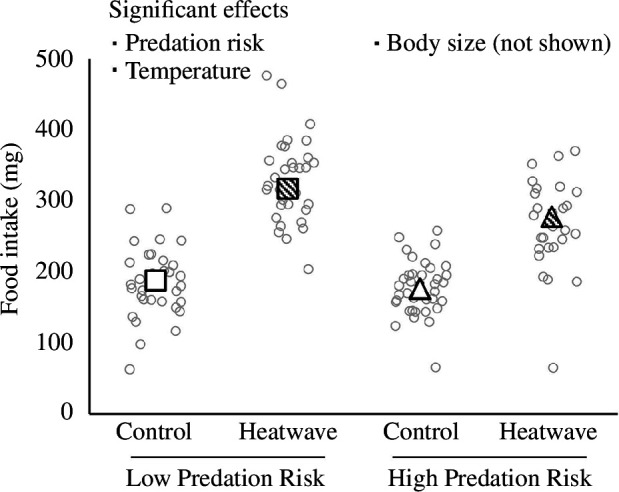
Effects of predation risk (low risk versus high risk) and temperature (control regime versus simulated heatwave) on food (dry cat food pellets) intake during early adulthood in female *G. lineaticeps* (*n* = 131). Summary values are shown as estimated marginal mean ± s.e.m. because initial (day 1) body mass was included as a covariate to account for differences in body size. Raw values are denoted by grey circle symbols.

**Figure 2.**
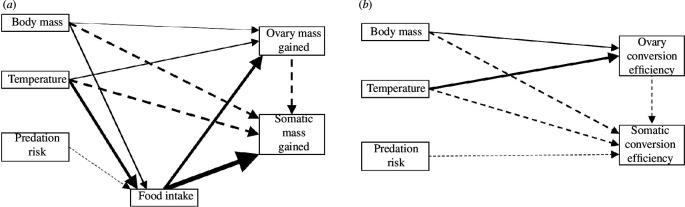
Final, most parsimonious path models characterizing the effects of predation risk (low risk versus high risk), temperature (control regime versus simulated heatwave) and body size on (*a*) estimated ovary and somatic tissue mass gained, and (*b*) the estimated conversion efficiencies of ingested food into ovary and somatic tissue mass during early adulthood in *G. lineaticeps* (*n* = 131). Positive effects are shown in solid paths, negative effects are shown in dashed paths and the thickness of paths indicate the strength of effects (i.e. the standardized regression coefficients or *β* weights). The initial, full path models are shown in the electronic supplementary material, figure S2.

**Figure 3.**
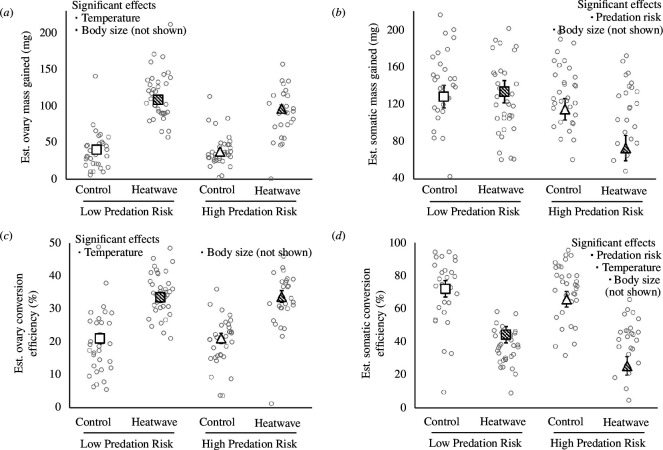
Effects of predation risk (low risk versus high risk) and temperature (control regime versus simulated heatwave) on the estimated amount of (*a*) reproductive tissue and (*b*) somatic tissues gained, and the efficiency by which ingested food was converted into (*c*) reproductive and (*d*) somatic tissues in *G. lineaticeps* (*n* = 131). Summary values are displayed as estimated marginal mean ± s.e.m. to account for body size. Raw values are denoted by grey circle symbols.

Food intake was higher for larger individuals, as well as those exposed to low predation risk and heatwave conditions (figures 1 and 2*a*; electronic supplementary material, tables S1 and S6). In turn, individuals that ate more food invested more into reproductive and somatic tissues, while larger individuals invested more into reproduction and less into soma (figure 2*a*; electronic supplementary material, tables S2, S3 and S6). Crickets exposed to heatwave also exhibited greater total reproductive investment while those investing less into reproduction exhibited greater total somatic investment (figure 2*a* and 3*a*; electronic supplementary material, tables S2 and S6). Predation risk reduced total somatic investment according to the linear mixed model (but not path) analyses (figures 2*a* and 3*b*; electronic supplementary material, table S3). There was a near-significant interaction between predation risk and temperature on total somatic investment (*p* = 0.064) where the somatic costs of predation risk were pronounced in heatwave conditions (figure 3*b*; electronic supplementary material, table S3). The conversion of food into ovary mass was greatest in larger individuals and those exposed to heatwave (figure 2*b*; electronic supplementary material, S2*c*; tables S4 and S7). By contrast, the conversion of food into somatic tissue was greatest in smaller individuals and those exposed to control temperature, as well as in those investing less into reproduction and those exposed to low predation risk (figures 2*b* and 3*d*; electronic supplementary material, tables S5 and S7). Our linear mixed models detected no significant interactive effects of temperature and predation on any measured variable (figures 1 and 3; electronic supplementary material, tables S1–S5).

## 4. Discussion

Features of environmental change, such as warming, can affect animals at several levels of biological organization—from shifting physiology and behaviour to changing biotic interactions [2,37,38]. The physiology, behaviour and interspecific interactions of animals can be linked to life-history strategies [16,37,39,40], which may be important to animals’ responses to ongoing environmental change [20–22]. Therefore, we used *G. lineaticeps* to investigate the interconnected roles of heat, predation risk and foraging on life-history strategy—specifically, the allocation of ingested resources to reproduction versus soma. Heatwave promoted feeding while predation risk reduced feeding (figures 1 and 2); however, both environmental factors independently prioritized allocation to reproduction at an expense to somatic tissue investment and there was a trend for heatwave and predation risk to exert synergistic costs to somatic investment (figures 2 and 3). Thus, combined exposure to both heatwaves and predation risk may entail significant costs to self-maintenance, which may impair key physiological processes.

Regarding our first aim (whether predation risk promotes a terminal investment or reproductive restraint strategy), we did not find support for either strategy because of predation risk because it did not affect reproductive investment, and it reduced investment into somatic tissue (figures 2 and 3). Although crickets exposed to predation risk reduced their food intake (figure 1), they maintained ovary mass investment by reducing investment into and allocation to somatic tissue (figures 2 and 3). With respect to our second aim (whether the expression of terminal investment or reproductive restraint was context-dependent), we did not detect any significant interactive effects of heatwave and predation risk on life-history strategy or resource acquisition (figures 1 and 3). Warmer temperatures reduce adult lifespan in ectotherms, including in *G. lineaticeps*, thereby reducing future reproductive opportunities [41,42], but resource acquisition and allocation by heatwave-exposed crickets tended to be either unaffected by predation risk or more (not less) sensitive to predation risk. Thus, heat-induced changes to foraging and to the prioritization of reproduction over soma were robust, even when ecological costs were high. Somatic tissue mass gained in our study was probably in the form of fat body, which is the major energy storage tissue in insects, and is also highly metabolic and important in immunity and detoxification [43]. Warmer temperatures promote immune function in *G. lineaticeps* and other *Gryllus* [44,45], but heat tends to amplify the costs of chemical stressors to insects [46]. Therefore, life-history strategies that prioritize reproductive allocation over soma may make insects particularly vulnerable to chemical pollution associated with environmental change.

For our third aim, we tested whether life-history strategy was affected by heatwave and predation risk directly or, rather, solely indirectly via changes in feeding. Heat directly promoted reproductive investment and reduced somatic investment, even after accounting for variation in food intake (figures 2 and 3). By contrast, predation risk did not alter investment into or allocation to reproduction, and it exerted mixed effects on soma—one path analysis indicated a direct effect (figure 2*b*) while the other demonstrated only an indirect effect that was mediated by feeding (figure 2*a*). Food was freely available in our study, and food intake was the strongest determinant of reproductive and somatic tissue investment (*β* weights of 0.66 and 1.07, respectively; figure 2*a*). Food availability is becoming more variable or even declining in some regions owing to climate change [47–49], which may have complex effects on insects. Warming generally promotes insect reproduction, but its benefits can depend on food availability (this study; [50]). Warming may also increase mortality through its positive effects on predation [8–10]. Therefore, future work should investigate the combined effects of predation (rather than predation risk), food availability and heatwaves at the population or community level given the critical importance of insects to the functioning of terrestrial ecosystems (reviewed in [51]).

In summary, we show that heatwave—an increasingly prevalent feature of climate change—strongly promoted terminal investment, even after accounting for resource acquisition (food intake) and when predation risk was high. Meanwhile, predation risk had weaker effects on the reproduction–soma trade-off. We encourage further investigations into the roles of heat and predation risk on the plasticity of life-history strategies. Our study manipulated these factors only during early adulthood and in an animal strongly motivated to invest in current reproduction. The expression of terminal investment and reproductive restraint may be context-dependent for other stressors (e.g. immune challenge [27,52])—thus, it is possible that older or other, longer-lived animals may exhibit different shifts in life-history strategy owing to exposure to heat and/or predation risk. Further, prolonged, developmental exposure to heat or predation risk may alter the dynamics of resource acquisition and allocation. Because we found that predation risk and heatwave altered investment into fitness-related traits, our work is an important step towards understanding fear ecology in a changing world.

## Data Availability

All data are freely available through FigShare (https://figshare.com/s/29c744e89ba17b5056dc). Electronic supplementary material is available online [51,53].
